# Aging and Prosociality

**DOI:** 10.1093/geroni/igaf122.1875

**Published:** 2025-12-31

**Authors:** Molly Lin, Helene Fung

## Abstract

Aging has been found to positively correlate with prosocial behaviors, such as charitable donations and volunteering. Older adults not only engage in more prosocial behavior but also derive greater benefits from it compared to activities focused on self-development. Socioemotional selectivity theory explains this by categorizing prosocial goal as one of the ‘emotionally meaningful goals’ that increase with age. Yet, questions remain unknown from this perspective about what makes prosociality so meaningful and significant to older adults, and whether positive emotions drive or result from older adults’ prosocial behaviors. This symposium addresses three questions: 1. WHETHER older adults are more prosocial than younger adults under any circumstances (i.e., what the boundaries of this age-related effect are), 2. WHY this is the case (i.e., what the underlying mechanisms are), and 3. HOW prosocial behavior can contribute to positive aging. Five presentations tap into these questions using theoretical, experimental, and ecological momentary approaches. They examined the relative importance of prosocial versus pro-closeness values, compared the factors associated with prosocial behaviors from adolescence to late adulthood, and explored the anti-loneliness effects of volunteering in older age. This symposium will shed light on future studies to further explore the mechanisms and outcomes of older adults’ prosocial behavior.

